# Body weight and blood pressure changes on dolutegravir‐, efavirenz‐ or atazanavir‐based antiretroviral therapy in Zimbabwe: a longitudinal study

**DOI:** 10.1002/jia2.26216

**Published:** 2024-02-08

**Authors:** Tinei Shamu, Matthias Egger, Tinashe Mudzviti, Cleophas Chimbetete, Justen Manasa, Nanina Anderegg

**Affiliations:** ^1^ Newlands Clinic Harare Zimbabwe; ^2^ Institute of Social and Preventive Medicine University of Bern Bern Switzerland; ^3^ Graduate School of Health Sciences University of Bern Bern Switzerland; ^4^ Centre for Infectious Disease Epidemiology and Research School of Public Health University of Cape Town Cape Town South Africa; ^5^ Population Health Sciences, Bristol Medical School University of Bristol Bristol UK; ^6^ Department of Pharmacy and Pharmaceutical Sciences University of Zimbabwe Harare Zimbabwe; ^7^ Innovation Hub University of Zimbabwe Harare Zimbabwe

**Keywords:** HIV, dolutegravir, blood pressure, hypertension, body weight, Zimbabwe

## Abstract

**Introduction:**

Dolutegravir (DTG) is widely used for antiretroviral therapy (ART). We compared weight and blood pressure trends and examined the association between high blood pressure and weight gain among people living with HIV (PLHIV) switching to or starting DTG‐based, efavirenz (EFV)‐based and ritonavir‐boosted atazanavir (ATV/r)‐based ART in Zimbabwe.

**Methods:**

PLHIV aged 18 years or older who started or switched to DTG, EFV or ATV/r‐based ART between January 2004 and June 2022 at Newlands Clinic in Harare, Zimbabwe, were eligible. Weight was measured at all visits (Seca floor scales); blood pressure only at clinician‐led visits (Omron M2 sphygmomanometer). We used Bayesian additive models to estimate trends in weight gain and the proportion with high blood pressure (systolic >140 mmHg or diastolic >90 mmHg) in the first 2 years after starting or switching the regimen. Finally, we examined whether trends in the proportion with high blood pressure were related to weight change.

**Results:**

We analysed 99,969 weight and 35,449 blood pressure records from 9487 adults (DTG: 4593; EFV: 3599; ATV/r: 1295). At 24 months after starting or switching to DTG, estimated median weight gains were 4.54 kg (90% credibility interval 3.88−5.28 kg) in women and 3.71 kg (3.07−4.45 kg) in men, around twice that observed for ATV/r and over four‐times the gain observed for EFV. Prevalence of high blood pressure among PLHIV receiving DTG‐based ART increased from around 5% at baseline to over 20% at 24 months, with no change in PLHIV receiving EFV‐ or ATV/r‐based ART. High blood pressure in PLHIV switching to DTG was associated with weight gain, with stronger increases in the proportion with high blood pressure for larger weight gains.

**Conclusions:**

Among PLHIV starting ART or switching to a new regimen, DTG‐based ART was associated with larger weight gains and a substantial increase in the prevalence of high blood pressure. Routine weight and blood pressure measurement and interventions to lower blood pressure could benefit PLHIV on DTG‐based ART. Further studies are needed to elucidate the mechanisms and reversibility of these changes after discontinuation of DTG.

## 1 INTRODUCTION

The integrase strand transfer inhibitor (INSTI) dolutegravir (DTG) in combination with tenofovir disoproxil fumarate and lamivudine has been endorsed by the World Health Organization (WHO) since 2018 as preferred first‐line antiretroviral therapy (ART) regimen for people living with HIV (PLHIV) initiating therapy, as well as offering an alternative second‐line regimen alongside an optimized nucleoside reverse transcriptase inhibitor (NRTI) [1]. In 2019, WHO recommended that switching of PLHIV with suppressed viral load from non‐nucleoside reverse transcriptase inhibitors (NNRTIs)‐based to DTG‐based ART should be considered, taking into account drug supply and patient choice [2].

Several studies found an association between INSTI‐based ART and weight gain [3, 4, 5, 6, 7, 8, 9]. DTG was associated with greater weight gain than other antiretroviral classes and older INSTIs like raltegravir [6]. Among PLHIV newly initiating ART or switching regimens following treatment failure, weight gain can be expected as part of the desired return‐to‐health phenomenon [10, 11]. However, among PLHIV transitioning from successful non‐DTG‐based ART to DTG for programmatic reasons, weight gain is an undesired effect that could herald an increasing cardiometabolic disease risk, including associated hypertension and chronic kidney disease [12]. Older cohort studies in the United States and Europe demonstrated the precedence of obesity before hypertension [13, 14, 15]. Mendelian randomization studies indicate a likely causal association [16], supported by research showing blood pressure improvements through non‐pharmacological or pharmacological weight reduction interventions [17, 18].

In Zimbabwe, an estimated 1.3 million people lived with HIV in 2022, with approximately 91% on ART [19]. In May 2019, Zimbabwe joined many other countries in transitioning PLHIV on first‐line ART from efavirenz (EFV)‐ to DTG‐based regimens, and favouring DTG for those initiating ART [20, 21]. Weight and blood pressure trends, and their association, are not well described for PLHIV across various ART regimens in low‐ and middle‐income countries. We compared weight and blood pressure trends among PLHIV starting or switching to DTG‐, EFV‐ and ritonavir‐boosted atazanavir (ATV/r)‐based ART and examined the association between high blood pressure and weight gain in a large ART programme in Zimbabwe.

## 2 METHODS

We analysed routine data of PLHIV attending the Newlands Clinic in Harare, Zimbabwe. After ART initiation, a clinician typically sees patients at weeks 4, 8, 12, 24, 36 and 48, then every 6 months unless they have comorbidities requiring closer monitoring or viral non‐suppression.

From 2013 to 2019, local ART guidelines recommended EFV‐based ART for first‐line treatment of HIV [22, 23]. Individuals who had started ART earlier (typically a nevirapine‐based regimen) were programmatically transitioned to EFV‐based regimens from 2013 onwards. Meanwhile, individuals requiring a switch to second‐line ART or already on ritonavir‐boosted lopinavir were initiated on or switched to ATV/r [22, 23, 24]. Since 2019, DTG was used both as a first‐line ART component at initiation and as a second‐line agent for those failing first‐line ART, typically EFV‐based. PLHIV who were virologically suppressed (predominantly on EFV‐based regimens) were programmatically transitioned to DTG over time.

Blood pressure measurement has been recommended at Newlands Clinic since 2014 but only became mandatory in 2018 as the clinic expanded its comprehensive care package to include a greater focus on screening and treatment of non‐communicable diseases. Nurses measured blood pressure using Omron M2 Intelli IT HEM‐7143T1‐EBK sphygmomanometers (OMRON Healthcare Europe B.V., Netherlands) after seating the client for at least 5 minutes. Cuff sizes (small: 17−22 cm, medium: 22−32 cm, large: 32−42 cm) were chosen depending on the middle upper arm circumference. Hypertension was diagnosed when the systolic or diastolic blood pressure was above 140 mmHg or above 90 mmHg, respectively, on 2 or more days [16]. Body weight was measured using Seca 750 floor scales (Seca, Germany) or Omron HBF‐214‐EBW scales after patients removed shoes, jackets and emptied pockets.

### 2.1 Inclusion criteria and definitions

We included all PLHIV aged 18 years or older who had ever started or switched to DTG, EFV‐ or ATV/r‐based ART from January 2004 until database closure (30 June 2022) and had provided informed written consent. Baseline was defined as the time of starting or switching ART. We extracted weight and blood pressure measurements recorded within the first 2 years after baseline and weight measurements from the year before baseline. PLHIV who did not have baseline‐ or post‐baseline measurements were excluded. We also excluded women pregnant at baseline or within the preceding year. Only weight measurements before pregnancy were included in women who became pregnant after baseline. We excluded blood pressure measurements in PLHIV diagnosed with hypertension at or before baseline, and those on antihypertensive treatment [25].

We examined both absolute and proportional weight change. Absolute weight change was defined as the difference in a weight measurement from the baseline value, while proportional weight change was the percentage change relative to the baseline value. We defined high blood pressure as systolic pressure ≥140 mmHg or diastolic pressure ≥90 mmHg. We grouped age at baseline into two categories, 18−39 and 40+ years, as they split the study population in approximately equal proportions and also define the categories for different approaches to hypertension screening [26]. Consistent with accepted nomenclature, baseline body mass index (BMI) was grouped into underweight (BMI <18.5 kg/m^2^), normal range (18.5 to <25 kg/m^2^) and overweight or obese (≥25 kg/m^2^) [27].

### 2.2 Statistical analysis

Analyses of weight trends were done separately for the period before and after baseline. For each period, we aggregated data in three different ways:

1. Treatment regimen (three groups), sex (two groups) and month (25 groups in the post‐baseline and 13 groups in the pre‐baseline period).
2. Treatment regimen, sex, month and baseline BMI group (three groups).
3. Treatment regimen, sex, month and age group (two groups).

We excluded aggregated data cells if they contained fewer than 10 weight measurements. For each of the remaining cells, we calculated the median absolute and proportional weight change. To capture the precision of medians, we weighted data cells by the number of observations in the cell divided by the average number of observations across all cells. We then fitted Bayesian weighted additive models to analyse monthly trends in the median absolute and proportional weight changes. We included covariates sex and treatment regimen as well as their interaction and smoothed monthly trends by sex and treatment regimen. We assumed weakly informative priors and fitted models (1) overall and stratified by (2) baseline BMI and (3) age group.

Using similar models, we estimated monthly trends in the proportion of PLHIV with high blood pressure after baseline. The main differences consisted in fitting an unweighted model but adding a random intercept for study participant to the model's covariate structure (as we used individual level instead of aggregate data) and using a binomial additive instead of a linear additive model.

Finally, to examine whether time trends in the proportion of high blood pressure were related to weight changes, we restricted blood pressure data to measurements with available weight change measurement at that time. We then fitted the binomial additive mixed model again, including proportional weight change as a (time‐varying) covariate.

#### 2.2.1 Sensitivity analyses

We did four sensitivity analyses. First, we fitted weight and blood pressure models stratified by the combination of BMI and age group. Second, instead of aggregate data on median weight changes, we used individual‐level weight data and estimated mean overall weight changes (adding a random intercept for study participant). Third, to account for potential errors in measurements due to natural fluctuation of blood pressure, we restricted blood pressure data to only include measurements if a previous one was reported at most 10 months earlier for the same individual. We then estimated trends in the proportion of PLHIV with hypertension defined as two consecutive high blood pressure measurements. Fourth, we restricted the weight analysis to individuals who had switched ART for programmatic reasons (excluding those who newly initiated and those switching post virologic failure) and who were not underweight—focusing on the group where weight gains are least desirable and not expected because of the “return‐to‐health” phenomenon.

We produced posterior predictive distributions for median absolute and proportional weight changes and the proportion with high blood pressure or hypertension across all combinations of covariates sex, treatment regimen and time. For the (time‐varying) weight‐adjusted blood pressure analysis, we predicted trends in the proportion with high blood pressure for three specific weight gain trajectories—constant monthly increases leading to a 0%, 10% and 20% increase in weight 2 years after baseline. Posterior predictive distributions for model fits with random effects were marginalized by integration over the distribution of random effects. We summarize estimated posterior predictive distributions with medians and 90% credible intervals (CrI). Additional details about statistical models, prior distributions and posterior predictions are available in Text S1.

### 2.3 Ethics

PLHIV enrolling at Newlands Clinic provided informed written consent for research use of routine care data under the International epidemiology Databases to Evaluate AIDS (IeDEA) collaboration [28], approved by the Medical Research Council of Zimbabwe (MRCZ No. A1336).

## 3 RESULTS

### 3.1 Study population

**Figure 1:**
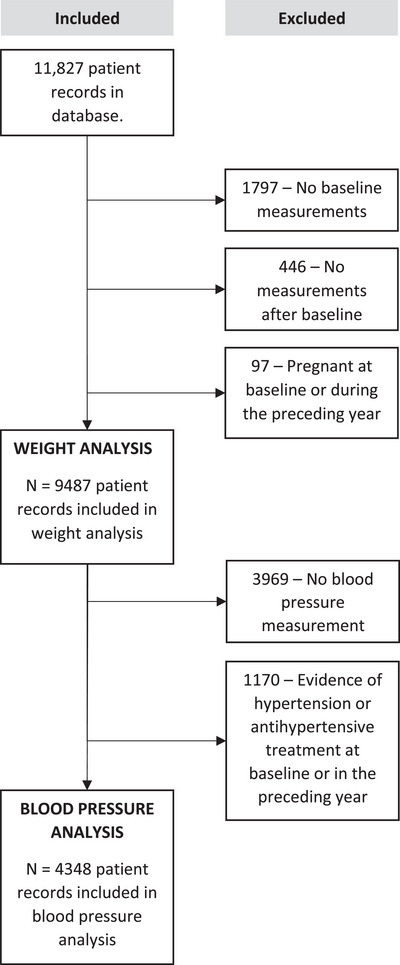
Flow chart of study participants included and excluded from the study and the different analyses.

We identified 11,827 records of individuals switching to or starting DTG, EFV or ATV/r. After excluding those without a weight measurement at or after baseline and pregnant women, we included 9487 PLHIV (80.2%, Figure 1). The 9487 PLHIV consisted of 6378 unique individuals, of whom 3339 contributed weight data to one regimen group, 2969 contributed weight data to two regimen groups (e.g. people who initially started with EFV and were later switched to DTG) and 70 contributed data to all three regimen groups (Table 1).

**Table 1:** Baseline characteristics of the 9487 study participants^a^ included in analyses

	DTG (*n* = 4593)	EFV (*n* = 3599)	ATV/r (*n* = 1295)
**Sex**			
Female	2985 (65.0%)	2359 (65.5%)	769 (59.4%)
Male	1608 (35.0%)	1240 (34.5%)	526 (40.6%)
**BMI (kg/m^2^)**			
Median (IQR)	23.5 (20.4−27.9)	23.5 (20.5−27.9)	22.0 (19.4−25.8)
<18.5	468 (10.2%)	374 (10.4%)	225 (17.4%)
≥18.5 to <25	2290 (49.9%)	1791 (49.8%)	692 (53.4%)
≥25	1835 (40.0%)	1434 (39.8%)	378 (29.2%)
**Age (years)**			
Median (IQR)	43 (33−50)	39 (32−47)	36 (23−45)
18−39 years	1814 (39.5%)	1800 (50.0%)	747 (57.7%)
40+ years	2779 (60.5%)	1799 (50.0%)	548 (42.3%)
**ART experience**			
Starting ART	321 (7.0%)	1413 (39.3%)	17 (1.3%)
Switching ART			
programmatically	4065 (88.5%)	2186 (60.7%)	0 (0%)
due to virologic failure	200 (4.4%)	0 (0%)	1278 (98.7%)
unknown reason	7 (0.2%)	0 (0%)	0 (0%)
Median (IQR) year of starting or switching ART	2019.9 (2019.7−2020.2)	2015.6 (2014.7−2015.8)	2015.8 (2013.7−2017.6)
**NRTI backbone**			
Abacavir + lamivudine	2 (<0.1%)	59 (1.6%)	465 (35.9%)
Zidovudine + lamivudine	1 (<0.1%)	232 (6.4%)	406 (31.4%)
Stavudine + lamivudine	−	112 (3.1%)	1 (0.1%)
Tenofovir disoproxil fumarate + lamivudine	4507 (98.1%)	3195 (88.8%)	421 (32.5%)
Tenofovir alafenamide fumarate + emtricitabine	83 (1.8%)	−	−
Other	−	1 (<0.1%)	2 (0.2%)
**Availability of measurements and inclusion in analysis**			
Weight measurement in the year before start/switch available	4306 (93.8%)	3235 (89.9%)	1159 (89.5%)
Blood pressure measurement in the 2 years after start/switch available	4125 (89.8%)	966 (26.8%)	427 (33.0%)
Included in blood pressure analysis (no diagnosis of hypertension, no antihypertensive treatment)	3181 (69.3%)	787 (21.9%)	380 (29.3%)
**Number of measurements per study participant** ^b^ **(mean [range])**			
Body weight after start/switch	7.2 (2.0−24.0)	13.0 (2.0−25.0)	15.5 (2.0−25.0)
Body weight before start/switch	5.2 (2.0−13.0)	6.1 (2.0−13.0)	9.0 (2.0−13.0)
Blood pressure measurement after start/switch	6.2 (1.0−23.0)	13.5 (1.0−24.0)	13.3 (1.0−25.0)

^a^ The 9487 PLHIV consisted of 6378 individuals, of whom 3339 contributed weight data to one regimen group only (1824 DTG only, 801 EFV only and 714 ATV/r only), 2969 contributed data to two regimen groups (2458 to both DTG and EFV, 241 to both ATV/r and DTG, and 270 to both EFV and ATV/r) and 70 contributed data to all three groups.

^b^ Including the measurement at baseline.

PLHIV were started with or switched to DTG in more recent years compared to the other two regimens, reflecting changes in the treatment guidelines (Table 1). Baseline BMI was similar for DTG and EFV, with around 10% categorized as underweight, 50% in the normal range and 40% overweight or obese, while the ATV/r group had a higher proportion (17.4%) of underweight PLHIV. The overall median age was 41 years but decreased from the DTG group (43 years) to the ATV/r group (36 years).

**Figure 2:**
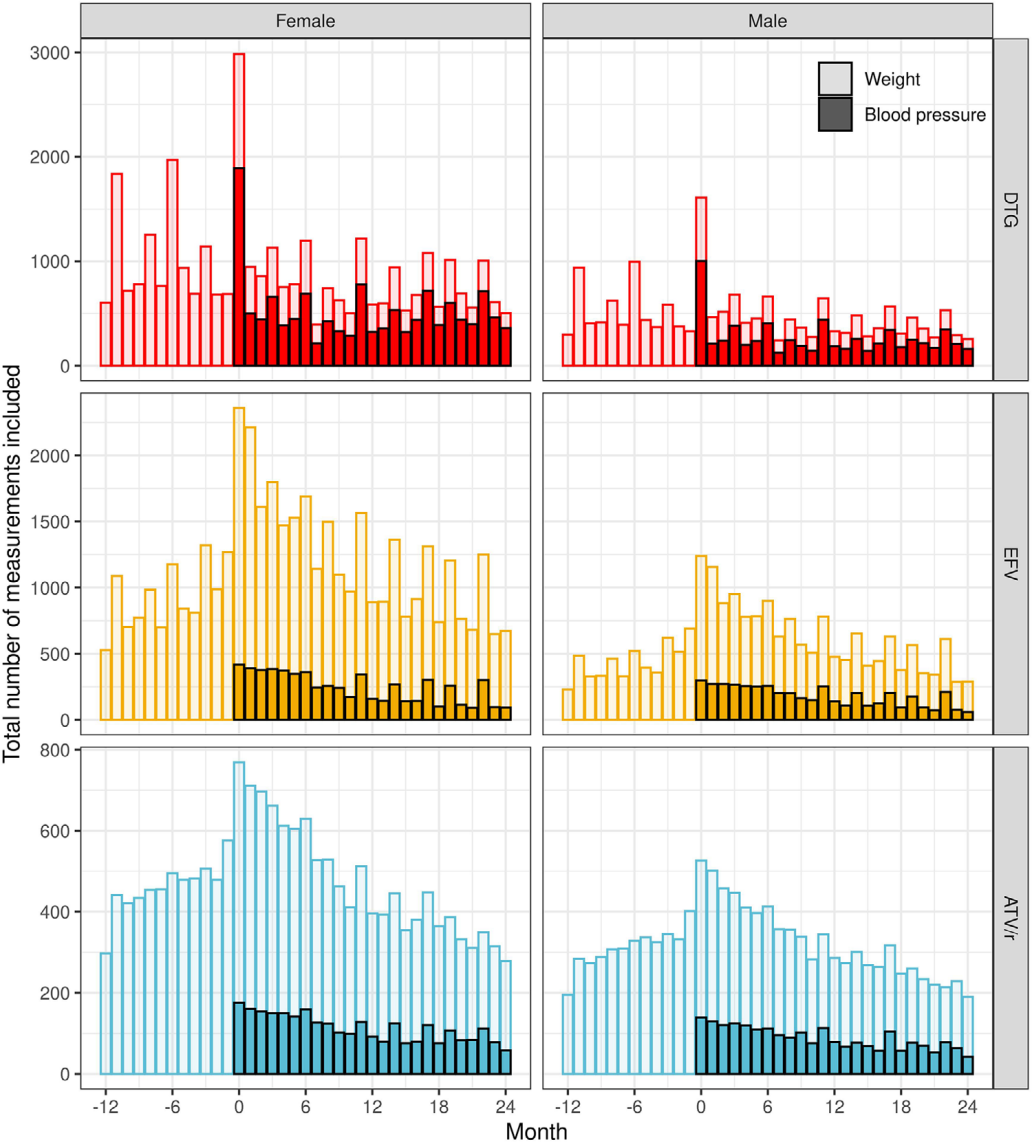
Total number of monthly body weight (more transparent) and blood pressure (less transparent) measurements by sex and treatment regimen. Month 0 corresponds to the time of start/switch. The period before start/switch is grey coloured to underline that study participants were not yet on the specific ART regimen.

Overall, 8700 (91.7%) PLHIV had a weight measurement before baseline, while only 5518 (58.2%) had a recorded blood pressure measurement available for review, with blood pressure measurements being more common for DTG (Table 1 and Figure 2). Four thousand three hundred and forty‐eight (45.8%) had no evidence of hypertension or antihypertensive use at baseline and were eligible for blood pressure analyses. Baseline characteristics of individuals with a weight measurement available before baseline were similar to those of all PLHIV. This was also the case for individuals eligible for blood pressure analyses, except for a younger age and more people newly initiating ART in the EFV group (Table S1).

### 3.2 Changes in body weight

Weight analyses included 9487 PLHIV (DTG: 4593, EFV: 3599, ATV/r: 1295) contributing 99,969 weight measurements after baseline (DTG: 33,057, EFV: 46,900, ATV/r: 20,012), and 52,615 weight measurements before baseline (DTG: 22,526, EFV: 19,686, ATV/r: 10,403). On an individual study participant level, absolute weight changes varied widely with some unrealistic gains or losses (Figure S1), with DTG‐based ART having the highest proportion experiencing any weight gain (Figure S2).

**Figure 3:**
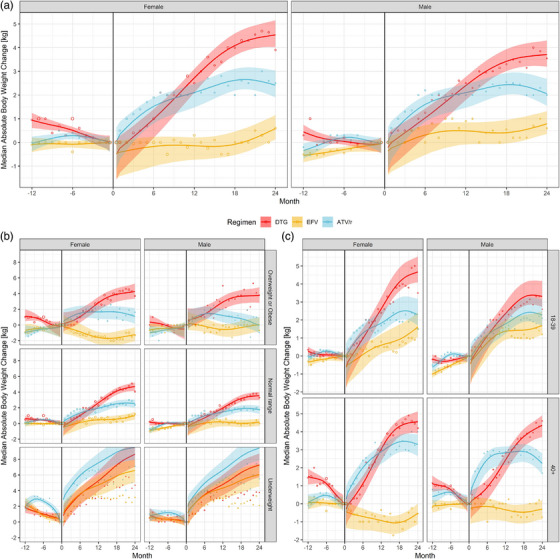
Median absolute weight change before and after starting or switching of ART by treatment regimen and sex. Results from Bayesian additive models fitted overall (A), and stratified by BMI baseline group (B) or age group (C). Month 0 corresponds to the time of start/switch. Medians of posterior predictive distributions are shown as solid lines, 90% credible intervals as shaded areas. The points correspond to the observed monthly medians in the data, the area of the points is proportional to the number of observations the medians were derived from. The period before start/switch is grey coloured to underline that study participants were not yet on the specific ART regimen.

When aggregated, the number of measurements per cell ranged from 23 to 2985 for the main analyses of weight trends. For the pre‐baseline period, estimated changes indicated constant or decreasing weight (Figure 3). Decreases were more pronounced for DTG, in overweight or obese PLHIV and older PLHIV. After baseline, DTG showed the largest increase in median weight, followed by ATV/r and EFV. At 24 months, estimated weight gain in females was 4.54 kg (90%‐CrI 3.88−5.28) for DTG, 2.42 kg (90%‐CrI 1.76−3.19) for ATV/r and 0.59 kg (90%‐CrI 0.00−1.28) for EFV, and in males 3.71 kg (90%‐CrI 3.07−4.45) for DTG, 2.06 kg (90%‐CrI 1.42−2.84) for ATV/r and 0.79 kg (90%‐CrI 0.22−1.44) for EFV (Figure 3). The differences between ART groups were greater for women than men and greater in PLHIV with a baseline BMI in the normal or overweight and obese range. For underweight PLHIV, median increases were similar across the three ART groups, but the model did not fit the observed medians well. For DTG and ATV/r, weight changes were similar among the two age groups, whereas, in the EFV group, only younger PLHIV showed an increase in median weight. Patterns in proportional weight changes were similar to those of absolute weight changes (Figure S3). In the DTG group, estimated median proportional weight increases surpassed 5% at 24 months in most BMI and age groups, whereas increases were less pronounced in the other ART groups.

### 3.3 Changes in blood pressure

**Figure 4:**
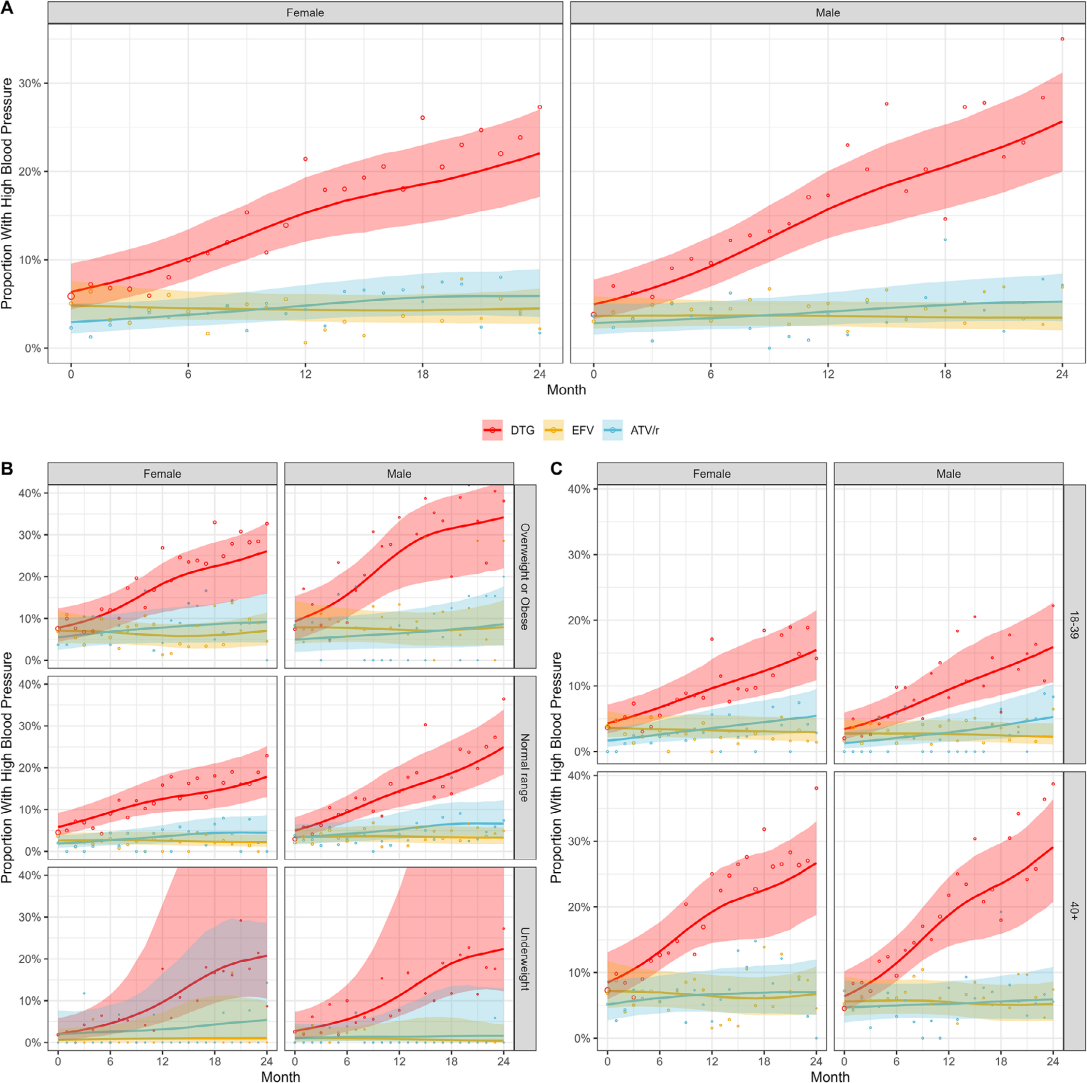
Proportion of study participants with high blood pressure after starting or switching of ART by treatment regimen and sex. Results from Bayesian binomial additive mixed models fitted overall (A), and stratified by BMI baseline group (B) or age group (C). Month 0 corresponds to the time of start/switch. Medians of marginalized posterior predictive distributions are shown as solid lines, 90% credible intervals as shaded areas. The points correspond to the observed proportions in the data, the area of the points is proportional to the number of blood pressure measurements.

Blood pressure analyses included 35,449 blood pressure measurements (DTG: 19,741, EFV: 10,635, ATV/r: 5073) from 4348 PLHIV (DTG: 3181, EFV: 787, ATV/r: 380). There was a clear increase in the observed proportion of PLHIV with high blood pressure in the DTG group, while for EFV and ATV/r, no such trend was observed (Figure S4). This was confirmed by model fits, where for DTG, the proportion with high blood pressure increased from an estimated 6.4% (90%‐CrI 4.4−9.6%) at baseline to 22.1% (90%‐CrI 17.1−27.0%) at 24 months in women, and from 4.9% (90%‐CrI 3.3−7.6%) to 25.7% (90%‐CrI 20.0−31.2%) in men (Figure 4). This increase was observed across all strata, with more pronounced increases in older ages and overweight or obese PLHIV.

### 3.4 Changes in blood pressure by changes in body weight

**Figure 5:**
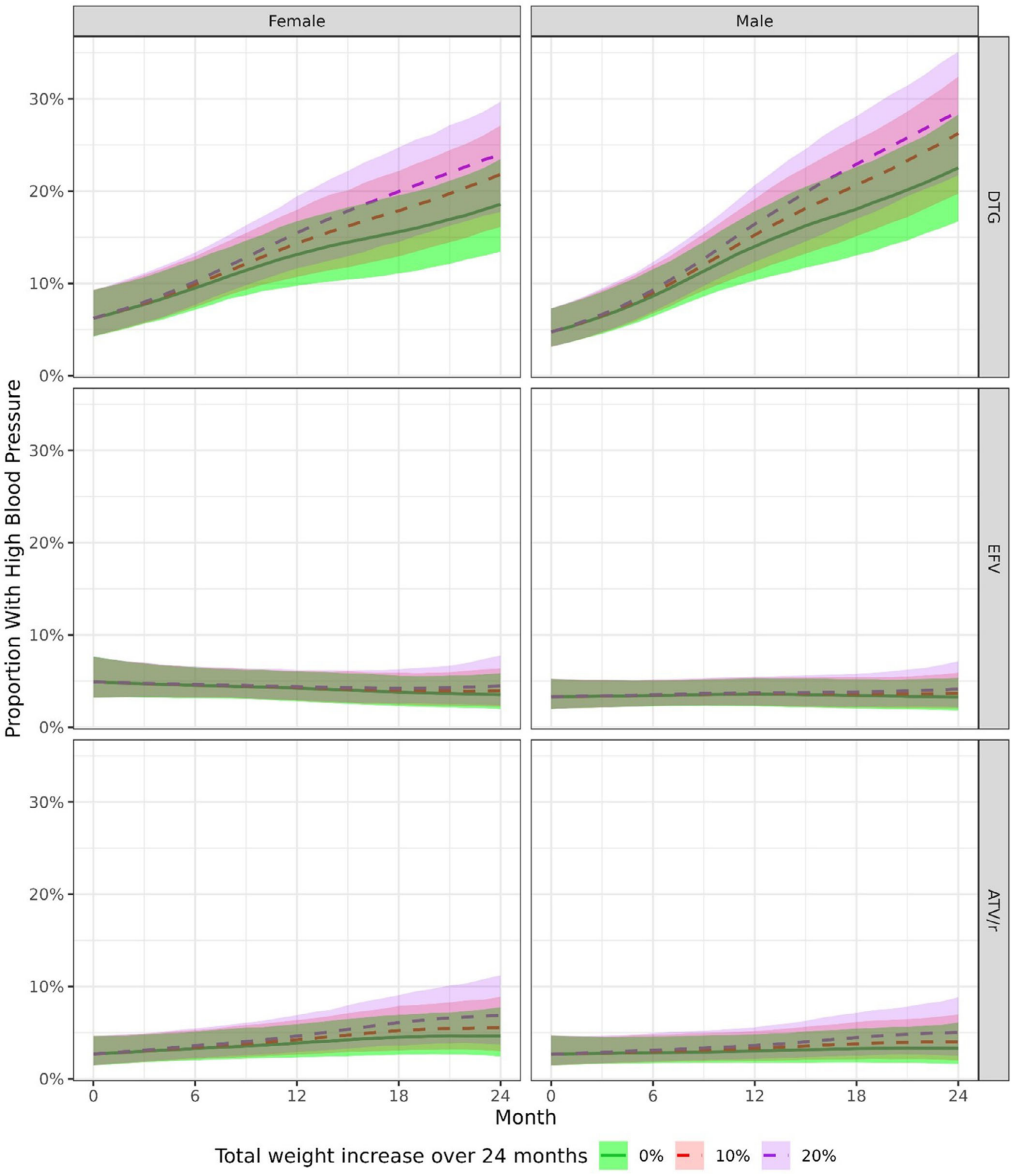
Proportion of participants with high blood pressure after starting or switching of ART by treatment regimen, sex and by monthly proportional weight gain. Results from Bayesian binomial additive mixed models. Predictions were derived by assuming a constant proportional monthly weight gain, adding up to a total of 0%, 10% or 20% increase in weight after 2 years. Month 0 corresponds to the time of start/switch. Medians of marginalized posterior predictive distributions are shown as solid (0% increase in weight over 2 years) or dashed (10%, 20% increase in weight over 2 years) lines, 90% credible intervals as shaded areas.

The model fit including proportional weight change as a time‐varying covariate showed that blood pressure trends in PLHIV switching to DTG correlated with weight gains, with stronger increases in the proportion with high blood pressure for larger weight gains (Figure 5). However, weight changes did not fully explain trends. Even for PLHIV without any weight gains after switching to DTG, the model predicted a substantial increase in the proportion with high blood pressure from 6.2% (90%‐CrI 4.2−9.3%) and 4.7% (90%‐CrI 3.2−7.3%) at baseline in females and males, respectively, to 18.6% (90%‐CrI 13.4−23.5%) and 22.5% (90%‐CrI 16.7−28.3%) 24 months later. This was observed across all BMI and age strata (Figures S5 and S6).

### 3.5 Sensitivity analyses

In the weight analysis stratified by BMI‐age‐group combinations, 15 out of 1368 aggregated cells with fewer than 10 observations were excluded. Still, some BMI‐age‐group combinations had poor model fit (Figure S7). DTG was associated consistently with the highest median weight gains in all strata except those categorized as underweight. The model fit to the individual study participant‐level weight data resembled the main analysis based on aggregate data, but estimated mean weight gains were generally larger, and the model did not fit the data well in the pre‐baseline period (Figure S8). Restricting analyses to non‐underweight individuals who programmatically switched ART included 1985 individuals for EFV, 3679 for DTG and none for ATV/r. DTG's estimated weight gains closely aligned with the main analysis (Figure S9). In the EFV group, results differed: while the main analysis indicated mostly stable weights with some increases in young individuals, the restricted analysis revealed a significant initial weight loss (followed by a slight recovery) which was consistent across most strata.

Blood pressure analyses stratified by BMI‐age‐groups were consistent with the main analysis (Figure S10). They indicated that for younger PLHIV starting or switching to DTG, the prevalence of high blood pressure mainly increased in those categorized as overweight or obese, while for older PLHIV, the increase was also apparent in the normal BMI group. The estimated proportions of PLHIV with hypertension (two consecutive high blood pressure measurements) were lower than the proportions based on single high measurements (Figure S11). However, DTG still showed a clear increase in the proportion with hypertension, while for EFV and ATV/r, the proportion remained constant.

## 4 DISCUSSION

In this large ART programme of the Newlands Clinic in Harare, Zimbabwe, starting or switching to DTG‐based ART was associated with a weight gain of approximately 4 kg over 2 years, while weight gains were lower for ATV/r and EFV‐based ART. For blood pressure, differences between treatment regimens were even more distinct. The proportions of PLHIV with high blood pressure remained stable in the EFV and ATV/r groups, while it increased by 15−20% in the first 2 years after starting or switching to a DTG‐based regimen.

The use of real‐world data, the large sample size and the careful statistical modelling, which included several sensitivity analyses, are strengths of this study. Our study also has limitations. First, unmeasured confounding is likely. The predominant use of ATV/r following virologic failure suggests that individuals who switched to this regimen might differ in aspects such as treatment adherence and related factors. In contrast, many participants were switched programmatically from EFV‐based to DTG‐based ART, contributing data to both groups in our analysis. This overlap in data enhances the comparability between groups. Nevertheless, we acknowledge that DTG was introduced later, post‐2019, while EFV was primarily used in earlier periods. Consequently, confounding factors, including the impact of the COVID‐19 pandemic and other unaccounted variables like dietary or physical activity changes over time, may partly account for the observed differences between the two treatment groups. Second, PLHIV on DTG‐containing regimens had more blood pressure measurements due to a 2018 policy change, making them mandatory. Before 2018, blood pressure checks were recommended but not compulsory. PLHIV starting or switching to EFV or ATV/r before 2018 may have had blood pressure measurements during visits, but the documentation was incomplete. We did observe some differences between those with and without measurements. For example, among those on EFV, individuals with blood pressure measurements tended to be younger. Therefore, while we are confident that the PLHIV on DTG with blood pressure measurements are a representative sample, we cannot completely rule out selection bias for the EFV‐ and ATV/r groups. Third, in these routine data, we observed some improbable body weight changes. We excluded obvious errors and computed medians to avoid undue outlier influence. Fourth, after disaggregating participants by baseline BMI, limited blood pressure records in the underweight category resulted in wide credible intervals, preventing firm conclusions. Lastly, instances of using the wrong cuff size, particularly for obese clients cannot be ruled out, despite all cuff sizes being available.

The greater weight gains for DTG, particularly in women, confirm previous findings [3, 4, 7, 8, 9, 10, 11, 29]. The differences between ART groups, where the NNRTI‐based regimen showed little weight gain, also align with previous work [11, 30]. In our cohort, almost all in the ATV/r group switched to second‐line ART post virologic failure, about half in the EFV group‐initiated ART, and in the DTG group, most were programmatically switched. Individuals in the first two groups are more likely to experience return‐to‐health weight gain than those in the DTG group. This is supported by the BMI‐stratified analysis, where PLHIV with low BMI gained substantial weight irrespective of regimen. For DTG, median proportional weight increased by over 5% in most BMI and age groups over 24 months. A study in middle‐aged adults found that a 5% weight gain over 5 years increased the risk of metabolic syndrome [31]. In our study, follow‐up was shorter, yet the median gain surpassed the 5% threshold, suggesting a potential risk of metabolic syndrome and cardiovascular disease for many PLHIV [32, 33].

The weight gain observed with DTG but not with EFV could be due to EFV‐associated toxicity rather than an undesired effect of DTG. Loss‐of‐function polymorphisms in CYP2B6 can result in higher EFV concentrations due to reduced clearance and metabolism of the drug, impairing weight gain [34, 35, 36]. Consequently, weight gain observed for DTG in this study could represent recovery from impaired weight gain due to the impact of such CYP2B6 polymorphisms. In a previous study, we found a relatively high prevalence of the CYP2B6 genotype in Zimbabwean PLHIV (minor allele frequencies c.516T [42%] and c.983C [9%]) [37]. Our sensitivity analysis supports this interpretation, focusing on non‐underweight individuals who programmatically switched their ART regimen. In this subgroup, we observed weight loss after switching to EFV‐based ART and similarly noted weight loss in individuals in the year before switching to DTG, with many likely receiving EFV during that period.

Men, older PLHIV and those overweight or obese experienced the highest increases in blood pressure. Although a single measurement might be unreliable due to fluctuations, we observed consistent trends for hypertension, defined as two consecutive high readings. Obesity and older age are known risk factors for hypertension [38, 39, 40, 41]. DTG‐based ART and male sex have also been linked to hypertension [42, 43, 44]. However, some studies found only small changes in blood pressure among PLHIV gaining weight on DTG‐based ART [45]. In this study, over a fifth in the DTG group had high blood pressure by month 24, compared to less than 10% at baseline.

Weight gain may directly increase blood pressure through pathways involving adipose tissue activation and the renin‐angiotensin‐aldosterone system [41]. However, weight change alone did not fully account for the association we found between ART regimen and blood pressure. Even among individuals on DTG with no weight gain, sensitivity analyses predicted a significant increase in the proportion with high blood pressure. While formal tests of mediation were not done, this increase is related to the concept of the direct effect of a regimen on blood pressure in mediation analysis. We will continue to follow these PLHIV and examine whether the high prevalence of hypertension is sustained.

## 5 CONCLUSIONS

In conclusion, we observed greater weight gain and an increase in the prevalence of high blood pressure among PLHIV starting or switching to DTG‐ compared to ATV/r‐ or EFV‐based ART. These findings underscore the need for weight and blood pressure monitoring among PLHIV receiving DTG‐based ART in resource‐limited settings. Further studies are urgently needed to clarify the causal pathways leading to the weight gain and blood pressure increase associated with DTG‐based ART, and to document any increase in metabolic or cardiovascular risk [45, 46].

## COMPETING INTERESTS

All authors declare no competing interests in this study.

## AUTHORS’ CONTRIBUTIONS

Conception and design: TS, ME and NA. Drafting of the article: TS, ME and NA. Critical revision of the article for important intellectual content: all authors. Final approval of the article: all authors. Provision of study materials or patients: TS and CC. Statistical expertise: NA. Obtaining of funding: NA and ME. Administrative, technical or logistic support: TS and CC. Collection and assembly of data: TS.

## FUNDING

This work was supported by the U.S. National Institutes of Health's National Institute of Allergy and Infectious Diseases (NIAID); the Eunice Kennedy Shriver National Institute of Child Health and Human Development (NICHD); the National Cancer Institute (NCI); the National Institute on Drug Abuse (NIDA); the National Heart, Lung, and Blood Institute (NHLBI); the National Institute on Alcohol Abuse and Alcoholism (NIAAA); the National Institute of Diabetes and Digestive and Kidney Diseases (NIDDK); and the Fogarty International Centre (FIC) (grant Award Number U01AI069924). NA and ME were supported by the Swiss National Science Foundation (grants P500PM_203010, 32FP30‐189498).

## DISCLAIMER

The content is solely the responsibility of the authors and does not necessarily represent the official views of the National Institutes of Health.

## Supporting information


**Table S1**. Comparison of baseline characteristics of study participants with and without any weight measurement before start/switch (top) and with and without any blood pressure measurement (bottom).
**Figure S1**. Distribution of (individual study participant‐level) absolute body weight changes over time in the year before and 2 years after starting/switching treatment regimens by sex and treatment regimen. Month 0 corresponds to the time of start/switch. The period before start/switch is grey coloured to underline that study participants were not yet on the specific ART regimen.
**Figure S2**. Distribution of monthly proportional weight changes (compared to the baseline weight) in study participants over time by treatment regimen and sex. The area below the dashed line correspond to the fraction of study participants that experienced weight gains, the area above the dashed line corresponds to the fraction that experienced constant weight or weight losses. Month 0 corresponds to the month of starting/switching treatment regimen.
**Figure S3**. Median proportional weight change before and after starting or switching of ART by treatment regimen and sex. Results from Bayesian additive models fitted overall (A), and stratified by BMI baseline group (B), or age group (C). Month 0 corresponds to the time of start/switch. Medians of posterior predictive distributions are shown as solid lines, 90% credible intervals as shaded areas. The points correspond to the observed monthly medians in the data, the area of the points is proportional to the number of weight measurements the median was derived from. The period before start/switch is grey coloured to underline that study participants were not yet on the specific ART regimen.
**Figure S4**. Observed monthly proportions of study participants with high and normal blood pressure after starting or switching of ART by treatment regimen and sex.
**Figure S5**. Proportion of study participants with high blood pressure after starting or switching of ART by treatment regimen, sex and by monthly proportional weight gain stratified by BMI baseline group. Results from Bayesian binomial additive mixed models. Predictions were derived by assuming a constant proportional monthly weight gain, adding up to a total of 0%, 10%, or 20% increase in weight after 2 years. Month 0 corresponds to the time of start/switch. Medians of marginalized posterior predictive distributions are shown as solid (0% increase in weight over 2 years) or dashed (10%, 20% increase in weight over 2 years) lines, 90% credible intervals as shaded areas.
**Figure S6**. Proportion of study participants with high blood pressure after starting or switching of ART by treatment regimen, sex and by monthly proportional weight gain stratified by baseline age group. Results from Bayesian binomial additive mixed models. Predictions were derived by assuming a constant proportional monthly weight gain, adding up to a total of 0%, 10%, or 20% increase in weight after 2 years. Month 0 corresponds to the time of start/switch. Medians of marginalized posterior predictive distributions are shown as solid (0% increase in weight over 2 years) or dashed (10%, 20% increase in weight over 2 years) lines, 90% credible intervals as shaded areas.
**Figure S7**. Median absolute weight change before and after starting or switching of ART by treatment regimen and sex. Results from Bayesian additive models stratified by combinations of baseline BMI and age groups. Medians of posterior predictive distributions are shown as solid lines, 90% credible intervals as shaded areas. The points correspond to the observed monthly medians in the data, the area of the points is proportional to the number of weight measurements the median was derived from. Monthly medians are only shown and included in the model fit if the data cells contained at least 10 observations. The period before start/switch is grey coloured to underline that study participants were not yet on the specific ART regimen.
**Figure S8**. Estimated mean absolute weight change before and after starting or switching of ART by treatment regimen and sex. Results from Bayesian additive mixed models fitted to the individual study participant‐level data. Month 0 corresponds to the time of start/switch. Estimated medians of posterior predictive distributions (of weight changes) are shown as solid line, 90% credible intervals as shaded areas. The points correspond to the observed monthly means in the data. The period before start/switch is grey coloured to underline that study participants were not yet on the specific ART regimen.
**Figure S9**. Median absolute weight change before and after starting or switching of ART by treatment regimen and sex. Results from Bayesian additive models fitted overall (A), and stratified by BMI baseline group (B), or age group (C) restricted to individuals who have switched ART due to programmatic reasons (excluding those who have switched due to virologic failure or have newly initiated ART) and who were not underweight. Month 0 corresponds to the time of start/switch. Medians of posterior predictive distributions are shown as solid lines, 90% credible intervals as shaded areas. The points correspond to the observed monthly medians in the data, the area of the points is proportional to the number of observations the medians were derived from. The period before start/switch is grey coloured to underline that study participants were not yet on the specific ART regimen.
**Figure S10**. Proportion of study participants with high blood pressure after starting or switching of ART by treatment regimen and sex. Results from Bayesian binomial additive mixed models stratified by combinations of baseline BMI and age group. Medians of marginalized posterior predictive distributions are shown as solid lines, 90% credible intervals as shaded areas. The points correspond to the observed monthly proportions in the data, the area of the points is proportional to the number of observations each month.
**Figure S11**. Comparison of the trends in the estimated proportion with hypertension (2 consecutive high blood pressure measurements, displayed in color) and the estimated proportion with high blood pressure (1 measurement only, displayed in gray).

## Data Availability

The data that support the findings of this study are available from the corresponding author upon reasonable request.
