# Supplementary material for: Body weight and blood pressure changes on dolutegravir‐, efavirenz‐ or atazanavir‐based antiretroviral therapy in Zimbabwe: a longitudinal study
Source: J Int AIDS Soc. 2024 Feb 8;27(2):e26216. doi: 10.1002/jia2.26216 (PMC10853595; doi:10.1002/jia2.26216)
Supplement: Supplementary file 1 — Table S1. Comparison of baseline characteristics of study participants with and without any weight measurement before start/switch (top) and with and without any blood pressure measurement (bottom). Figure S1. Distribution of (individual study participant‐level) absolute body weight changes over time in the year before and 2 years after starting/switching treatment regimens by sex and treatment regimen. Month 0 corresponds to the time of start/switch. The period before start/switch is grey coloured to underline that study participants were not yet on the specific ART regimen. Figure S2. Distribution of monthly proportional weight changes (compared to the baseline weight) in study participants over time by treatment regimen and sex. The area below the dashed line correspond to the fraction of study participants that experienced weight gains, the area above the dashed line corresponds to the fraction that experienced constant weight or weight losses. Month 0 corresponds to the month of starting/switching treatment regimen. Figure S3. Median proportional weight change before and after starting or switching of ART by treatment regimen and sex. Results from Bayesian additive models fitted overall (A), and stratified by BMI baseline group (B), or age group (C). Month 0 corresponds to the time of start/switch. Medians of posterior predictive distributions are shown as solid lines, 90% credible intervals as shaded areas. The points correspond to the observed monthly medians in the data, the area of the points is proportional to the number of weight measurements the median was derived from. The period before start/switch is grey coloured to underline that study participants were not yet on the specific ART regimen. Figure S4. Observed monthly proportions of study participants with high and normal blood pressure after starting or switching of ART by treatment regimen and sex. Figure S5. Proportion of study participants with high blood pressure after starting or switching of A [file JIA2-27-e26216-s001.docx]

**Supplementary Table S1.** Comparison of baseline characteristics of study participants with and without any weight measurement before start/switch (top) and with and without any blood pressure measurement (bottom).

|  | **Measurement available/included in analyses** | | |  | **No measurement available/excluded from analyses** | | |
| --- | --- | --- | --- | --- | --- | --- | --- |
|  | **DTG** | **EFV** | **ATV/r** |  | **DTG** | **EFV** | **ATV/r** |
| **Weight before start/switch** |  |  |  |  |  |  |  |
| **Total study participants** | 4306 | 3235 | 1159 |  | 287 | 364 | 136 |
| **Sex** |  |  |  |  |  |  |  |
| Female | 2841 (66.0%) | 2142 (66.2%) | 689 (59.4%) |  | 144 (50.2%) | 217 (59.6%) | 80 (58.8%) |
| Male | 1465 (34.0%) | 1093 (33.8%) | 470 (40.6%) |  | 143 (49.8%) | 147 (40.4%) | 56 (41.2%) |
| **BMI [kg/m2]** |  |  |  |  |  |  |  |
| Median (IQR) | 23.6 (20.5-27.9) | 23.6 (20.6-28.0) | 22.1 (19.5-25.8) |  | 22.7 (19.7-26.0) | 22.7 (19.5-27.2) | 21.6 (18.5-25.4) |
| <18.5 | 429 (10.0%) | 310 (9.6%) | 191 (16.5%) |  | 39 (13.6%) | 64 (17.6%) | 34 (25.0%) |
| >18.5 to <25 | 2135 (49.6%) | 1622 (50.1%) | 626 (54.0%) |  | 155 (54.0%) | 169 (46.4%) | 66 (48.5%) |
| >25 | 1742 (40.5%) | 1303 (40.3%) | 342 (29.5%) |  | 93 (32.4%) | 131 (36.0%) | 36 (26.5%) |
| **Age [years]** |  |  |  |  |  |  |  |
| Median (IQR) | 43 (33-50) | 40 (32-47) | 36 (23-45) |  | 37 (29-46) | 36 (29-45) | 39 (25-48) |
| 18-39 years | 1649 (38.3%) | 1581 (48.9%) | 678 (58.5%) |  | 165 (57.5%) | 219 (60.2%) | 69 (50.7%) |
| 40+ years | 2657 (61.7%) | 1654 (51.1%) | 481 (41.5%) |  | 122 (42.5%) | 145 (39.8%) | 67 (49.3%) |
| **ART experienced** |  |  |  |  |  |  |  |
| Starting ART | 155 (3.6%) | 1139 (35.2%) | 2 (0.2%) |  | 166 (57.8%) | 274 (75.3%) | 15 (11.0%) |
| Switching ART | 4151 (96.4%) | 2096 (64.8%) | 1157 (99.8%) |  | 121 (42.2%) | 90 (24.7%) | 121 (89.0%) |
|  |  |  |  |  |  |  |  |
| **Blood pressure after start/switch** |  |  |  |  |  |  |  |
| **Total study participants** | 3181 | 787 | 380 |  | 1412 | 2812 | 915 |
| **Sex** |  |  |  |  |  |  |  |
| Female | 2060 (64.8%) | 462 (58.7%) | 218 (57.4%) |  | 925 (65.5%) | 1897 (67.5%) | 551 (60.2%) |
| Male | 1121 (35.2%) | 325 (41.3%) | 162 (42.6%) |  | 487 (34.5%) | 915 (32.5%) | 364 (39.8%) |
| **BMI [kg/m2]** |  |  |  |  |  |  |  |
| Median (IQR) | 23.2 (20.2-27.4) | 23.0 (20.5-27.5) | 21.4 (19.1-24.7) |  | 24.3 (20.8-28.5) | 23.6 (20.6-28.0) | 22.3 (19.5-26.3) |
| <18.5 | 345 (10.8%) | 81 (10.3%) | 69 (18.2%) |  | 123 (8.7%) | 293 (10.4%) | 156 (17.0%) |
| >18.5 to <25 | 1638 (51.5%) | 421 (53.5%) | 221 (58.2%) |  | 652 (46.2%) | 1370 (48.7%) | 471 (51.5%) |
| >25 | 1198 (37.7%) | 285 (36.2%) | 90 (23.7%) |  | 637 (45.1%) | 1149 (40.9%) | 288 (31.5%) |
| **Age [years]** |  |  |  |  |  |  |  |
| Median (IQR) | 42 (34-48) | 37 (30-43) | 37 (27-44) |  | 46 (IQR: 23-54) | 40 (IQR: 33-48) | 36 (IQR: 22-46) |
| 18-39 years | 1275 (40.1%) | 496 (63.0%) | 223 (58.7%) |  | 539 (38.2%) | 1304 (46.4%) | 524 (57.3%) |
| 40+ years | 1906 (59.9%) | 291 (37.0%) | 157 (41.3%) |  | 873 (61.8%) | 1508 (53.6%) | 391 (42.7%) |
| **ART experienced** |  |  |  |  |  |  |  |
| Starting ART | 236 (7.4%) | 787 (100.0%) | 0 (0.0%) |  | 85 (6.0%) | 626 (22.3%) | 17 (1.9%) |
| Switching ART | 2945 (92.6%) | 0 (0.0%) | 380 (100.0%) |  | 1327 (94.0%) | 2186 (77.7%) | 898 (98.1%) |

**Supplementary Figure S1.** Distribution of (individual study participant-level) absolute body weight changes over time in the year before and 2 years after starting/switching treatment regimens by sex and treatment regimen. Month 0 corresponds to the time of start/switch. The period before start/switch is grey coloured to underline that study participants were not yet on the specific ART regimen.

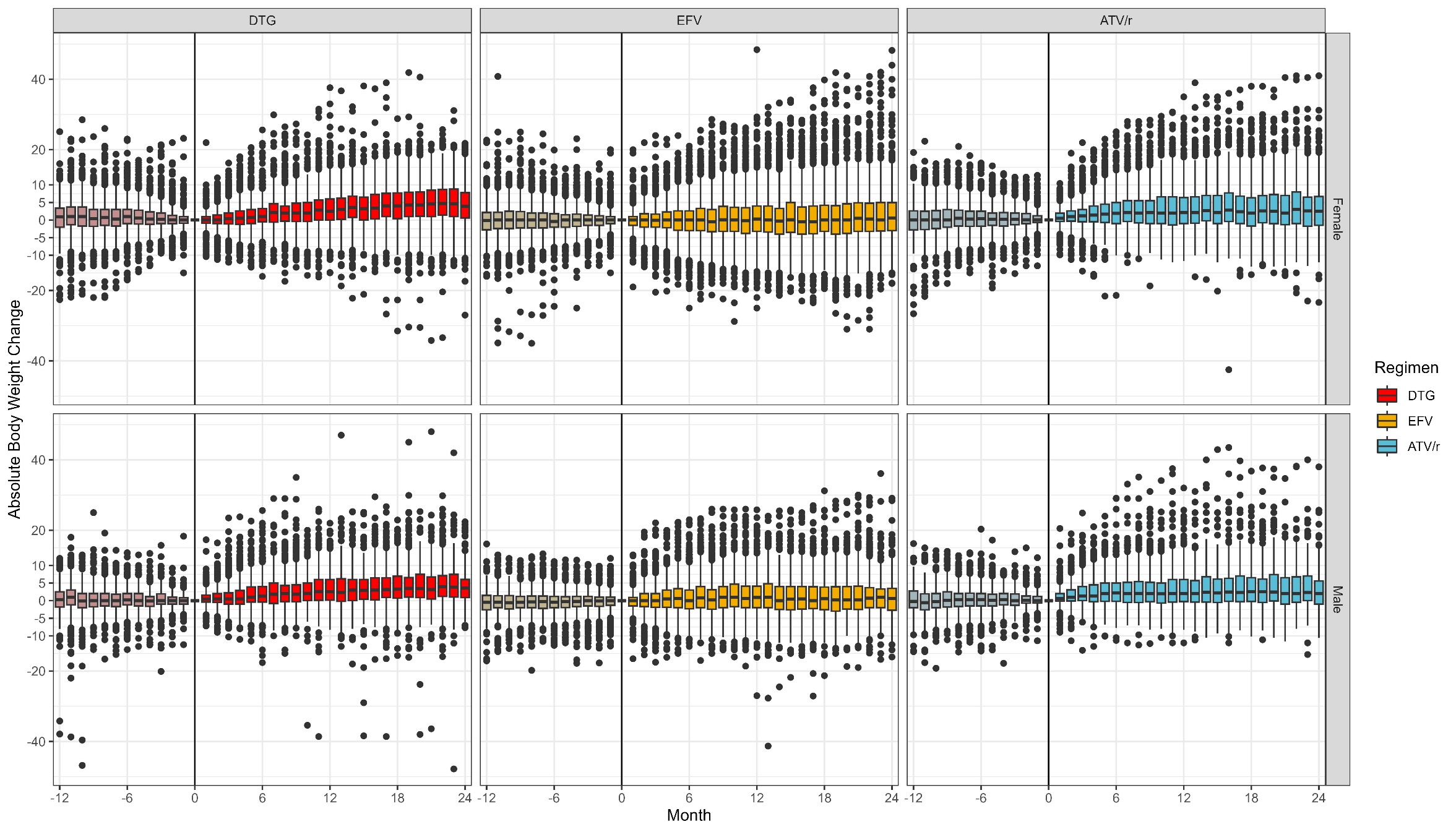


**Supplementary Figure S2.** Distribution of monthly proportional weight changes (compared to the baseline weight) in study participants over time by treatment regimen and sex. The area below the dashed line correspond to the fraction of study participants that experienced weight gains, the area above the dashed line corresponds to the fraction that experienced constant weight or weight losses. Month 0 corresponds to the month of starting/switching treatment regimen.

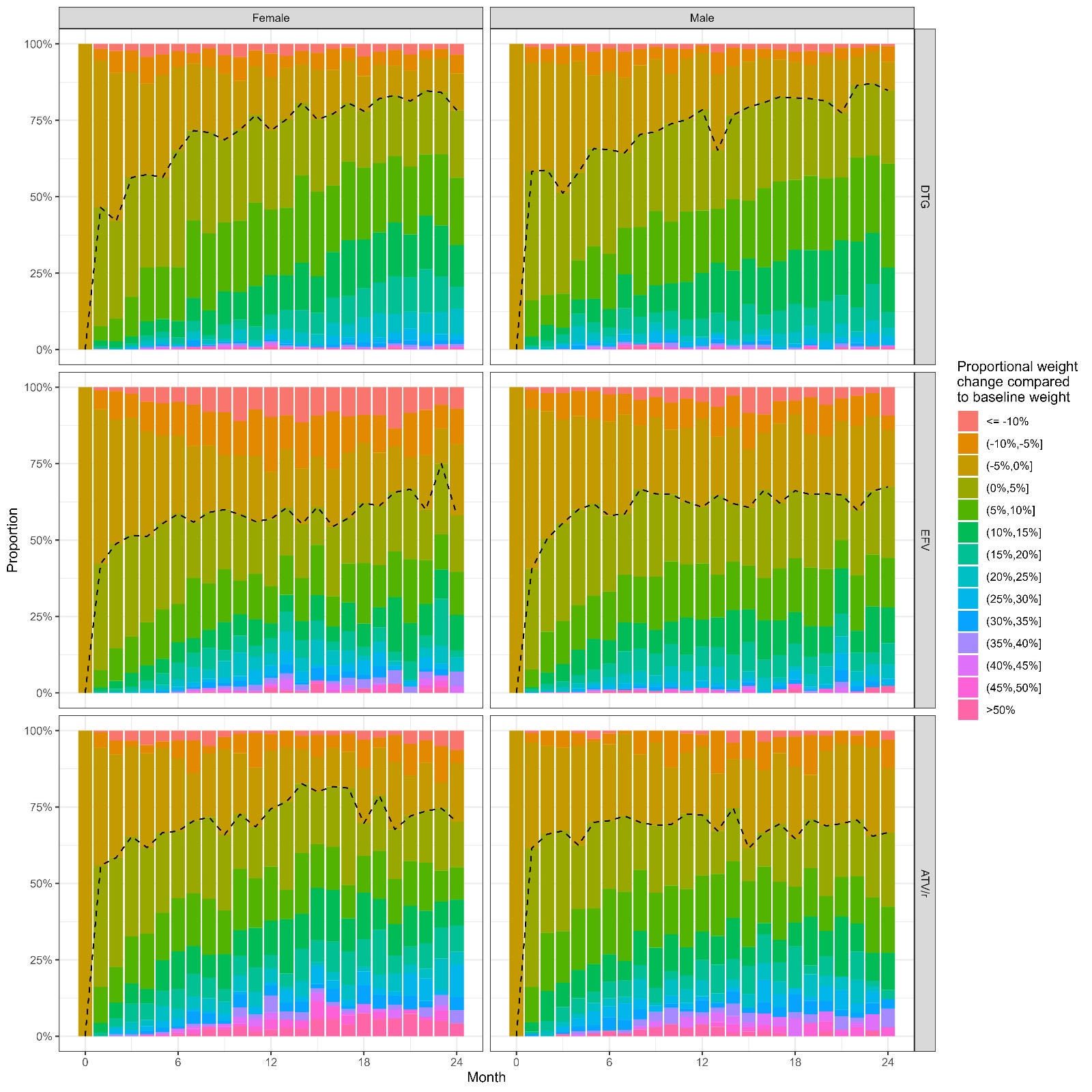


**Supplementary Figure S3.** Median proportional weight change before and after starting or switching of ART by treatment regimen and sex. Results from Bayesian additive models fitted overall (A), and stratified by BMI baseline group (B), or age group (C). Month 0 corresponds to the time of start/switch. Medians of posterior predictive distributions are shown as solid lines, 90% credible intervals as shaded areas. The points correspond to the observed monthly medians in the data, the area of the points is proportional to the number of weight measurements the median was derived from. The period before start/switch is grey coloured to underline that study participants were not yet on the specific ART regimen.

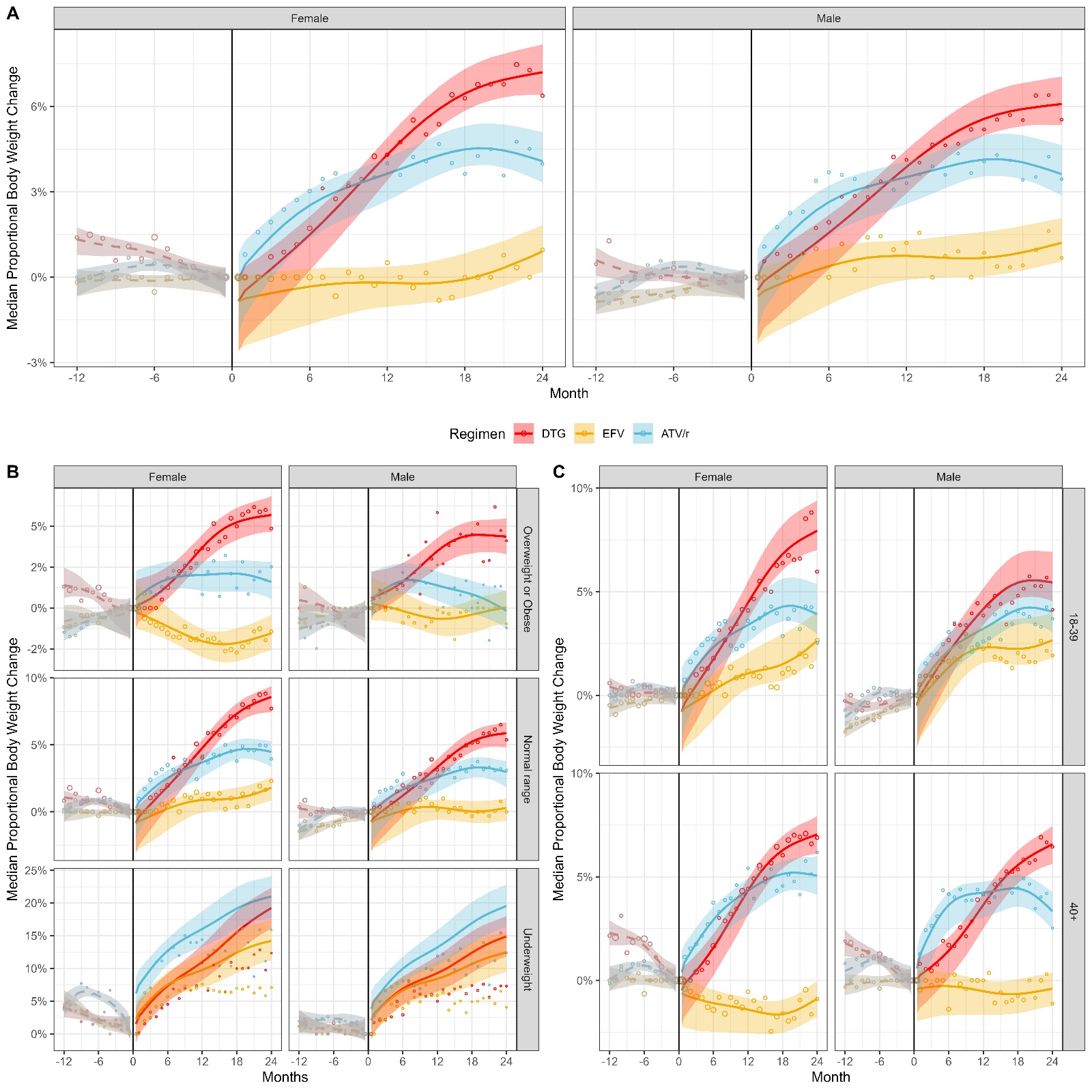


**Supplementary Figure S4.** Observed monthly proportions of study participants with high and normal blood pressure after starting or switching of ART by treatment regimen and sex.


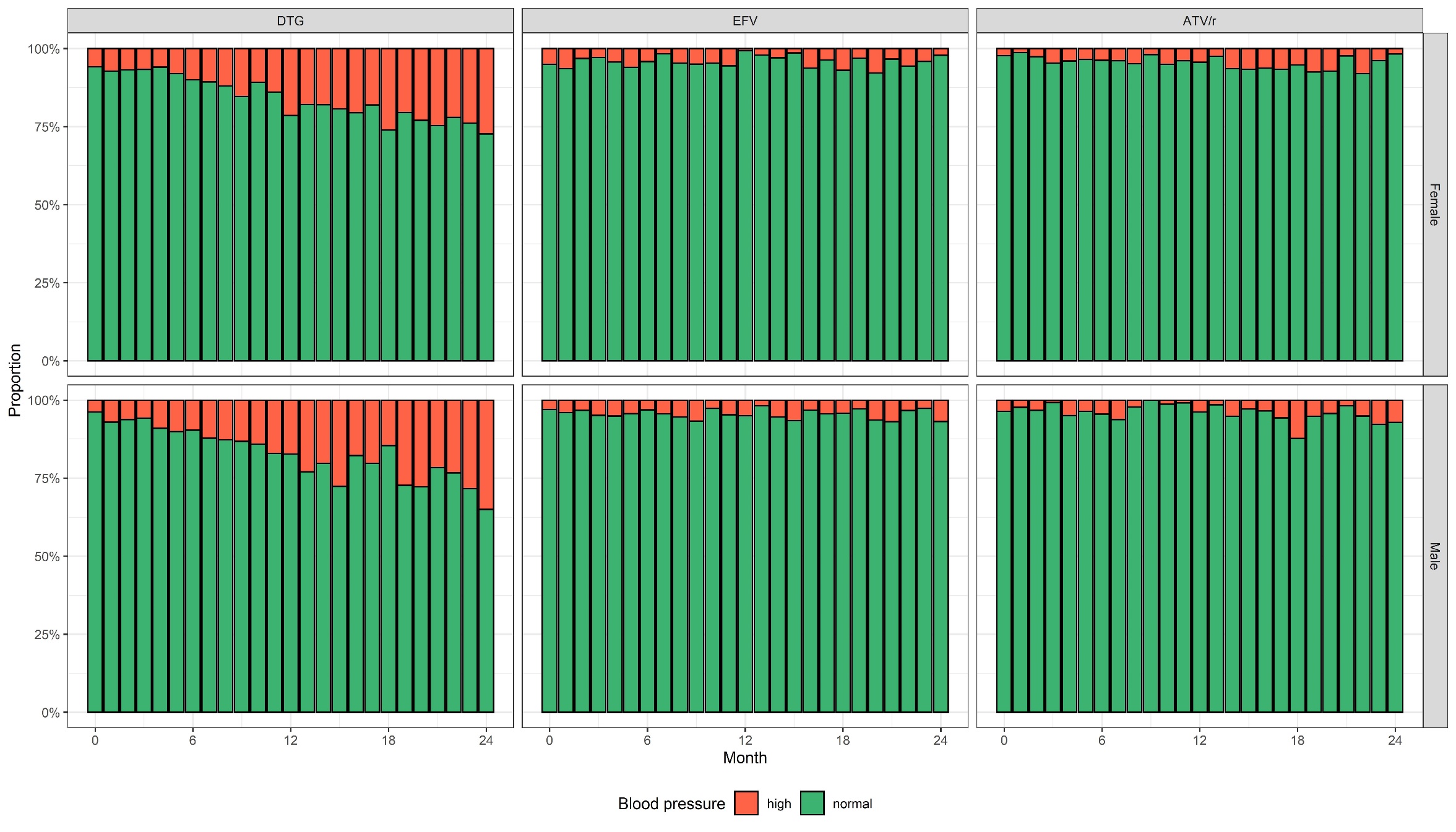


**Supplementary Figure S5.** Proportion of study participants with high blood pressure after starting or switching of ART by treatment regimen, sex and by monthly proportional weight gain stratified by BMI baseline group. Results from Bayesian binomial additive mixed models. Predictions were derived by assuming a constant proportional monthly weight gain, adding up to a total of 0%, 10%, or 20% increase in weight after 2 years. Month 0 corresponds to the time of start/switch. Medians of marginalized posterior predictive distributions are shown as solid (0% increase in weight over 2 years) or dashed (10%, 20% increase in weight over 2 years) lines, 90% credible intervals as shaded areas.

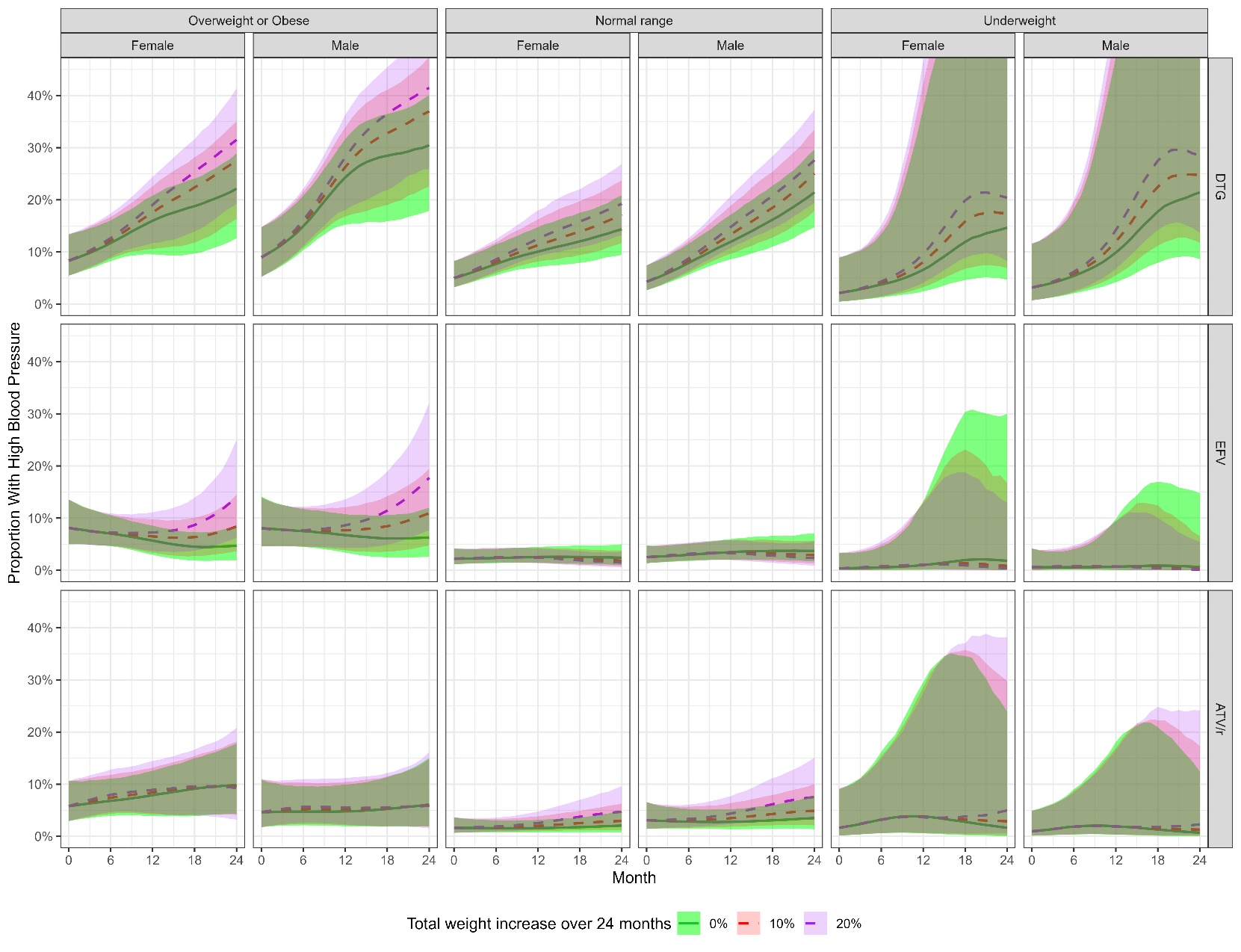


**Supplementary Figure S6.** Proportion of study participants with high blood pressure after starting or switching of ART by treatment regimen, sex and by monthly proportional weight gain stratified by baseline age group. Results from Bayesian binomial additive mixed models. Predictions were derived by assuming a constant proportional monthly weight gain, adding up to a total of 0%, 10%, or 20% increase in weight after 2 years. Month 0 corresponds to the time of start/switch. Medians of marginalized posterior predictive distributions are shown as solid (0% increase in weight over 2 years) or dashed (10%, 20% increase in weight over 2 years) lines, 90% credible intervals as shaded areas.


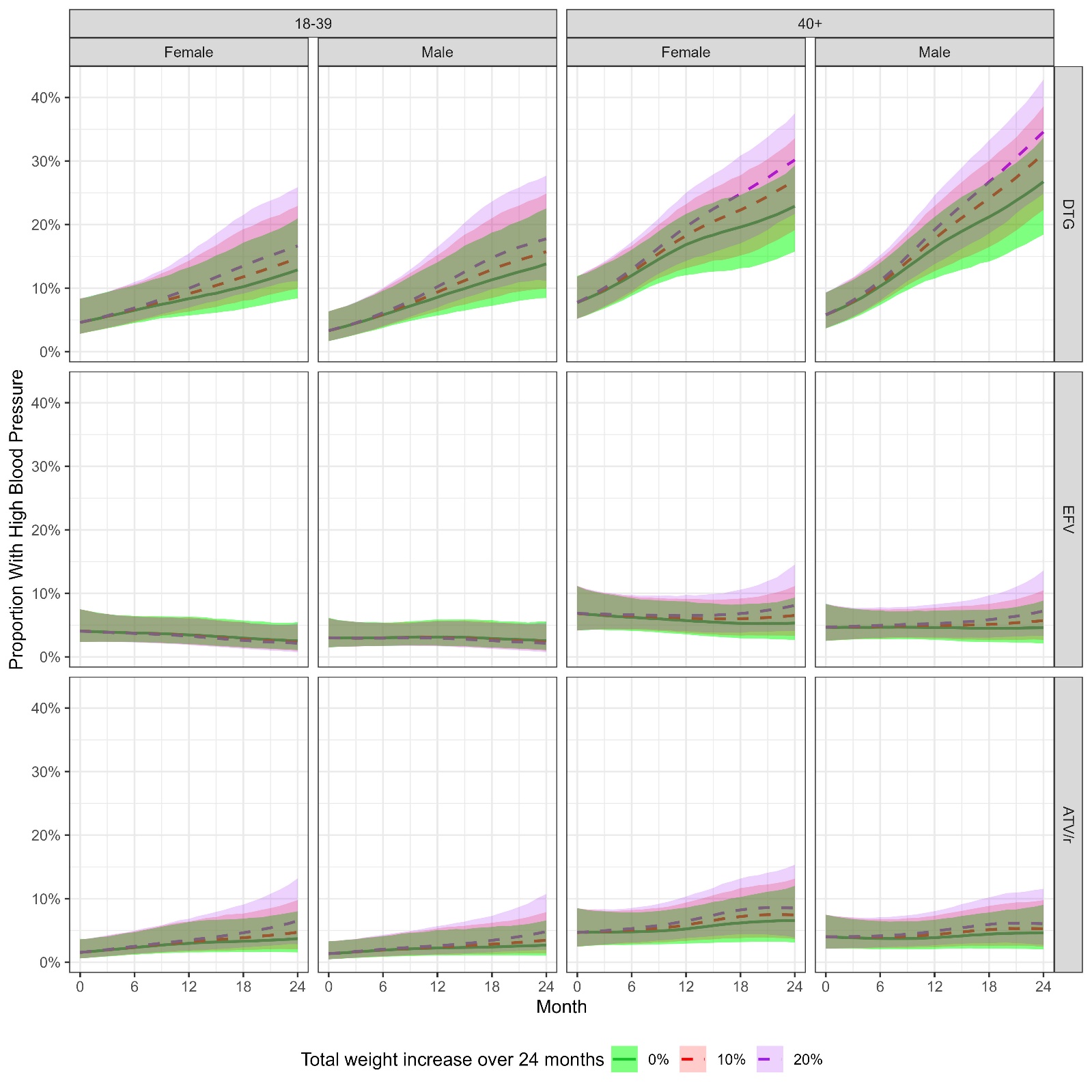


**Supplementary Figure S7.** Median absolute weight change before and after starting or switching of ART by treatment regimen and sex. Results from Bayesian additive models stratified by combinations of baseline BMI and age groups. Medians of posterior predictive distributions are shown as solid lines, 90% credible intervals as shaded areas. The points correspond to the observed monthly medians in the data, the area of the points is proportional to the number of weight measurements the median was derived from. Monthly medians are only shown and included in the model fit if the data cells contained at least 10 observations. The period before start/switch is grey coloured to underline that study participants were not yet on the specific ART regimen.


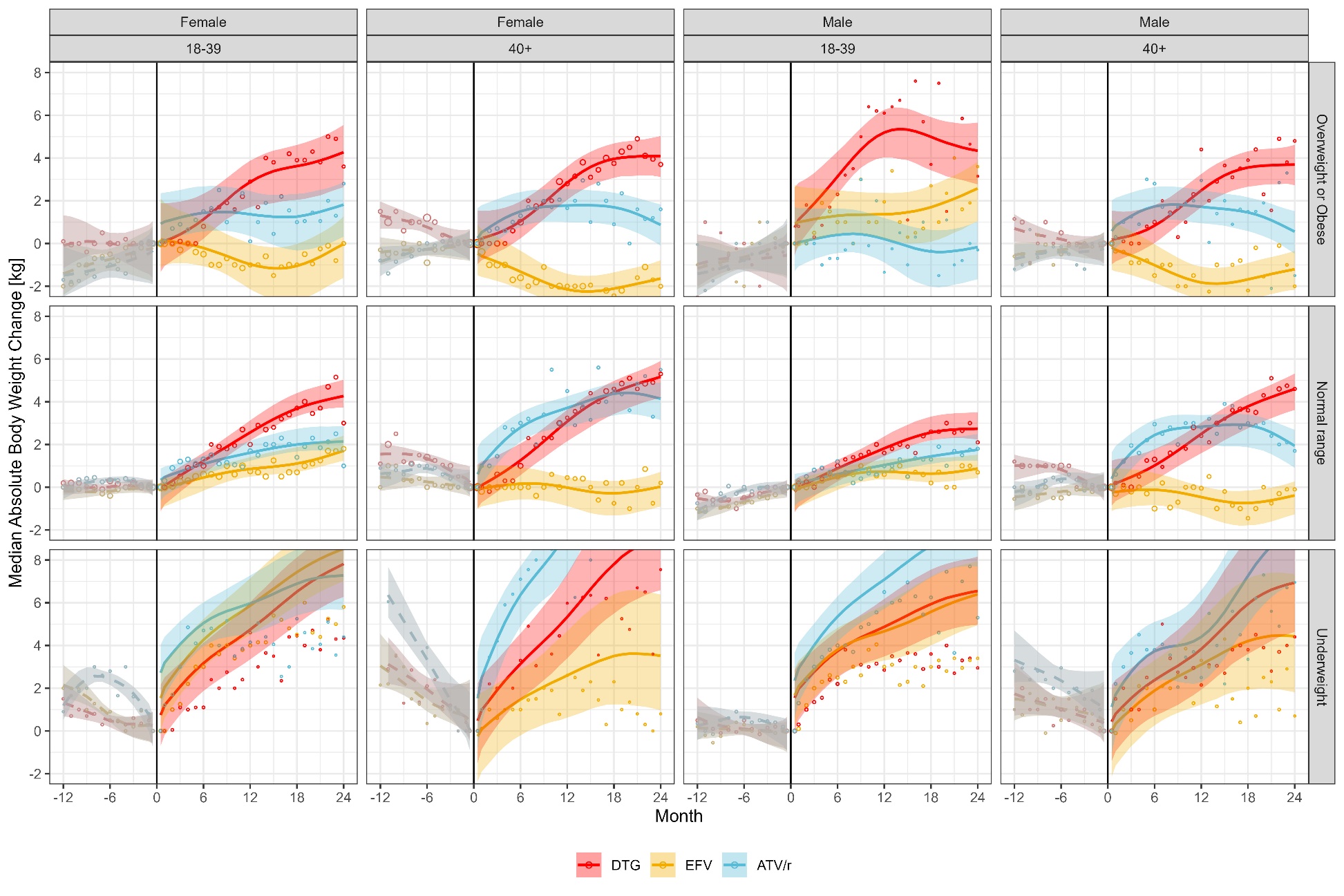


**Supplementary Figure S8.** Estimated mean absolute weight change before and after starting or switching of ART by treatment regimen and sex. Results from Bayesian additive mixed models fitted to the individual study participant-level data. Month 0 corresponds to the time of start/switch. Estimated medians of posterior predictive distributions (of weight changes) are shown as solid line, 90% credible intervals as shaded areas. The points correspond to the observed monthly means in the data. The period before start/switch is grey coloured to underline that study participants were not yet on the specific ART regimen.


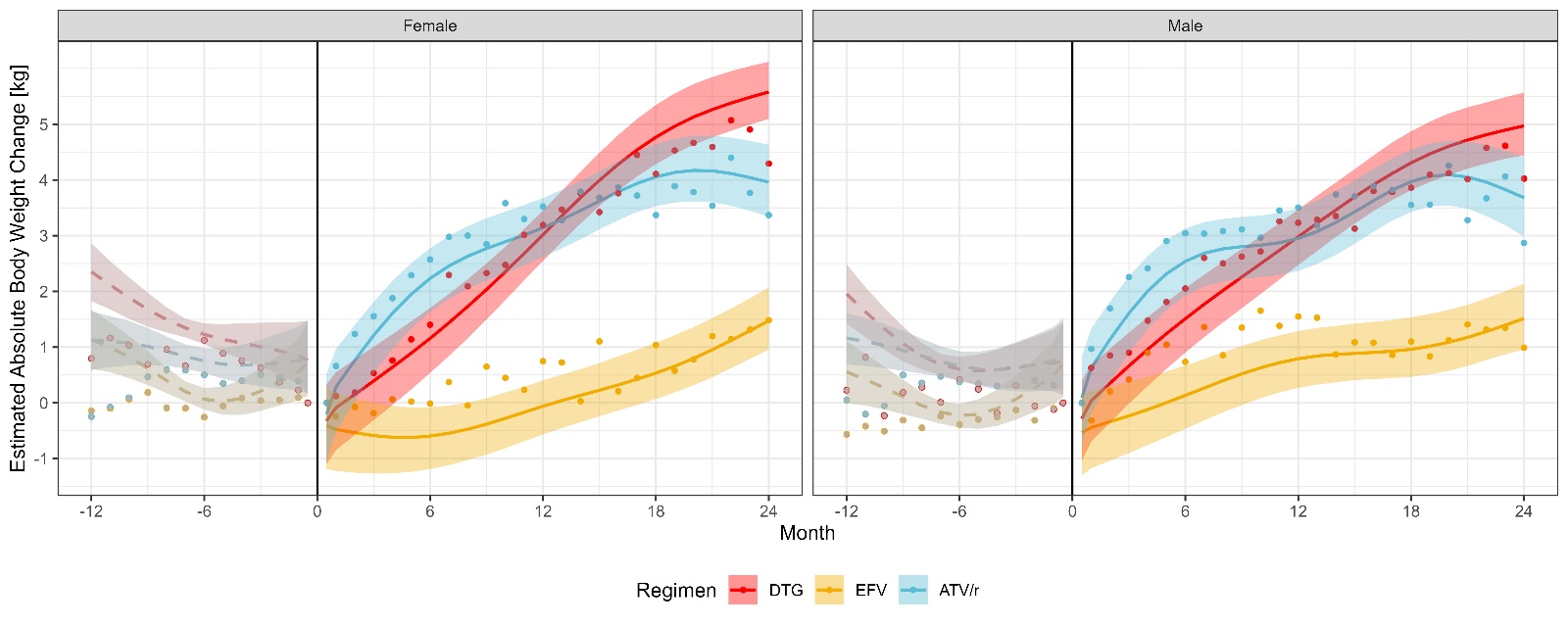


**Supplementary Figure S9.** Median absolute weight change before and after starting or switching of ART by treatment regimen and sex. Results from Bayesian additive models fitted overall (A), and stratified by BMI baseline group (B), or age group (C) restricted to individuals who have switched ART due to programmatic reasons (excluding those who have switched due to virologic failure or have newly initiated ART) and who were not underweight. Month 0 corresponds to the time of start/switch. Medians of posterior predictive distributions are shown as solid lines, 90% credible intervals as shaded areas. The points correspond to the observed monthly medians in the data, the area of the points is proportional to the number of observations the medians were derived from. The period before start/switch is grey coloured to underline that study participants were not yet on the specific ART regimen.

**
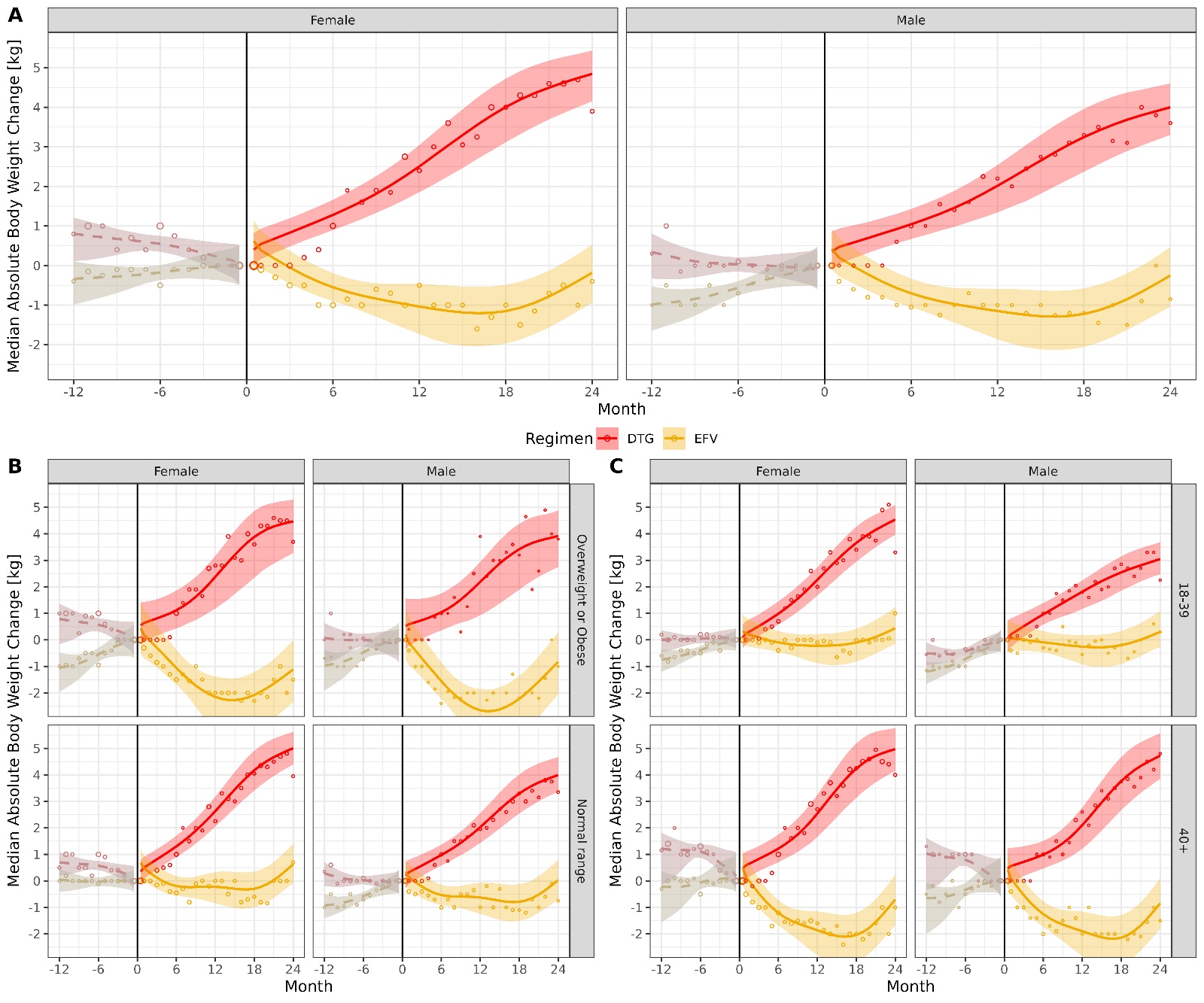
**

**Supplementary Figure S10.** Proportion of study participants with high blood pressure after starting or switching of ART by treatment regimen and sex. Results from Bayesian binomial additive mixed models stratified by combinations of baseline BMI and age group. Medians of marginalized posterior predictive distributions are shown as solid lines, 90% credible intervals as shaded areas. The points correspond to the observed monthly proportions in the data, the area of the points is proportional to the number of observations each month.


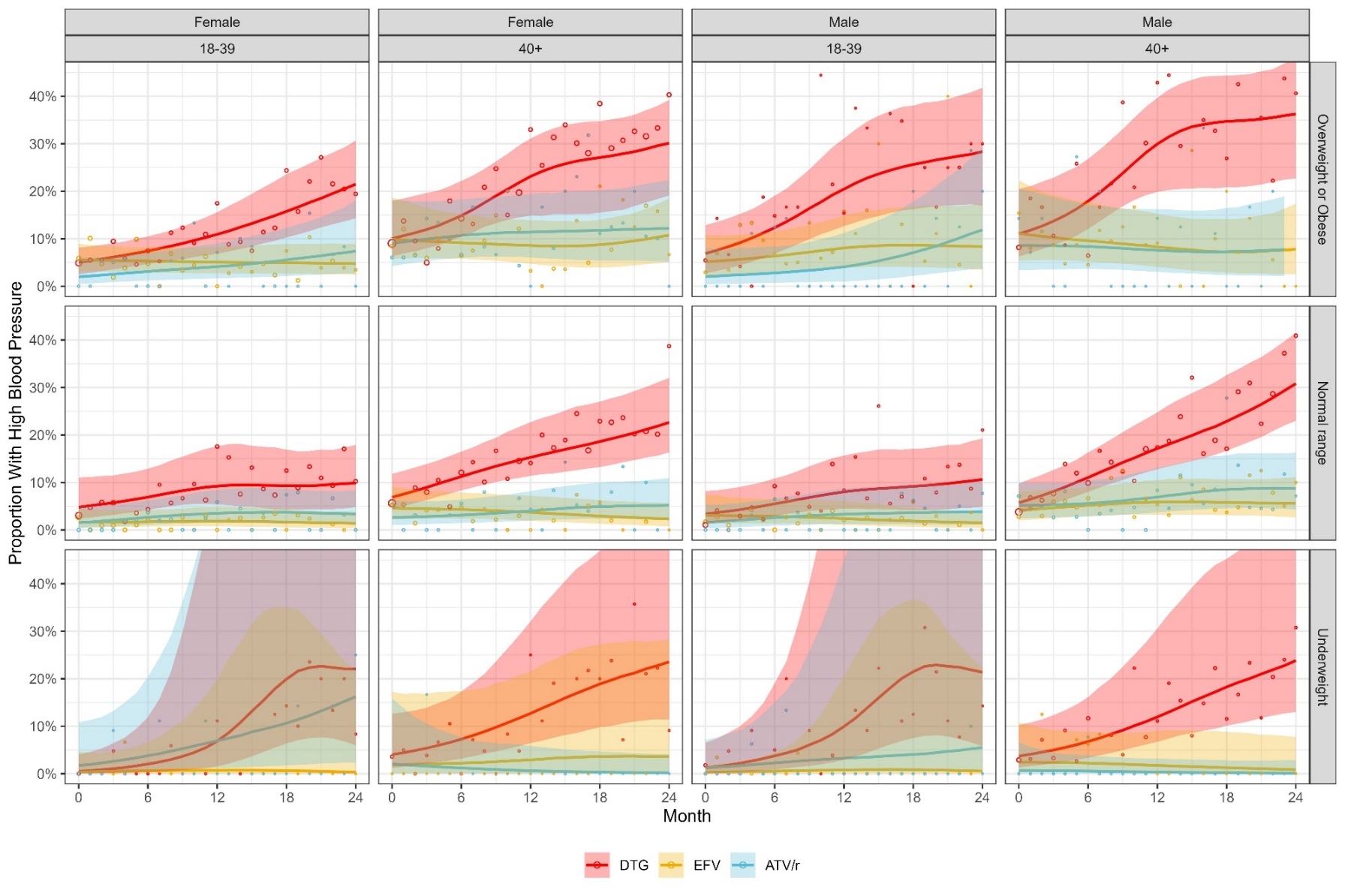


**Supplementary Figure S11.** Comparison of the trends in the estimated proportion with hypertension (2 consecutive high blood pressure measurements, displayed in color) and the estimated proportion with high blood pressure (1 measurement only, displayed in gray).

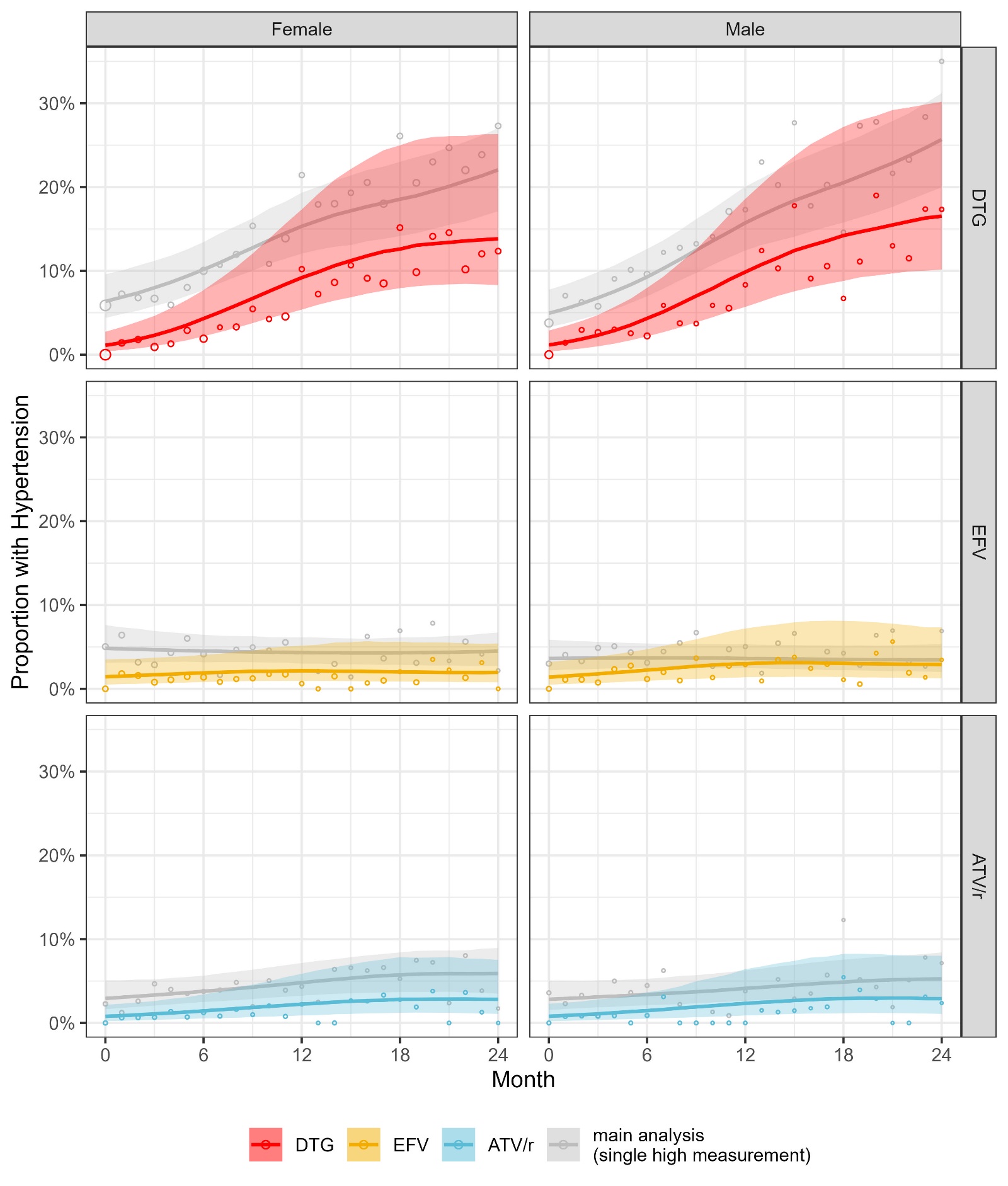


**Supplementary Text S1.** Information about model structure, fits, parameters, and predictions.

For this study, we used the R-package “rstanarm” and fitted the following models as described (simplified) below:

1. Trends in median absolute and proportional body weight changes (based on aggregate data):

stan_gamm4(median_weight_change (absolute or proportional) ~ Sex * ART_regimen + t2(Month, bs = “tp”) + t2(Month, ART_regimen, bs = c(“tp”, “fs”)) + t2(Month, ART_regimen, Sex, bs = c(“tp”, “fs”, “fs”)),

weights = weigths_accuracy_of_median, ….)

1. Trends in high blood pressure (based on individual study participant-level data):

stan_gamm4(high_blood_pressure ~ Sex * ART_regimen + t2(Month, bs = “tp”) + t2(Month, ART_regimen, bs = c(“tp”, “fs”)) + t2(Month, ART_regimen, Sex, bs = c(“tp”, “fs”, “fs”)),

random = ~(1|id), family = binomial, …)

1. Trends in high blood pressure accounting for proportional weight changes (based on individual study participant-level data):

stan_gamm4(high_blood_pressure ~ Sex * ART_regimen + proportional_weight_change*ART_regimen + t2(Month, bs = “tp”) + t2(Month, ART_regimen, bs = c(“tp”, “fs”)) + t2(Month, ART_regimen, Sex, bs = c(“tp”, “fs”, “fs”)) + t2(Month, ART_regimen, proportional_weight_change, bs = c(“tp”, “fs”, “tp”)),

random = ~(1|id), family = binomial, …)

1. Trends in mean body weight changes (based on individual study participant-level data):

stan_gamm4(weight_change ~ Sex * ART_regimen + t2(Month, bs = “tp”) + t2(Month, ART_regimen, bs = c(“tp”, “fs”)) + t2(Month, ART_regimen, Sex, bs = c(“tp”, “fs”, “fs”)),

random = ~(1|id), …)

1. Trends in hypertension (based on individual study participant-level data):

stan_gamm4(hypertension ~ Sex * ART_regimen + t2(Month, bs = “tp”) + t2(Month, ART_regimen, bs = c(“tp”, “fs”)) + t2(Month, ART_regimen, Sex, bs = c(“tp”, “fs”, “fs”)), random = ~(1|id), family = binomial, …)

Models A, B and C were fitted to the overall data (1), and then stratified by baseline BMI group (2) and baseline age group (3) (sensitivity analysis models D and E were only fitted without stratification). Weight models B and D were fitted separately to the period BEFORE start/switch and AFTER start/switch (blood pressure models B, C, and E were only fitted to the period AFTER start switch).

For the models we assumed the following weakly prior distributions for parameters:

- Intercept ~normal(location = 0, scale = 2.5)
- Coefficients: ~normal(location = 0, scale = 2.5)
- Auxiliary (sigma): ~exponential(rate = 1).
- For models with random intercept (Model B, C, D, E): Covariance: ~ decov(reg. = 1, conc. = 1, shape = 1, scale = 1)

We produced posterior predictions of median absolute and proportional weight changes (model A), mean absolute weight changes (model D), and the proportion with high blood pressure (model B) and hypertension (model E), for all combinations of covariates sex, treatment regimen and time. For model C, we predicted trends in the proportion of patients with high blood pressure for all different sex-regimen-time combinations, each combined with 3 specific weight gain trajectories over two years. More specifically, we predicted trends for patients who had a 0% (no change), 10%, and 20% increase in weight over two years. There are several ways on how to reach these increases. For example, a 20% increase in weight after two years can be reached by having no change in the first 23 months but then a large 20% increase in weight in the last month, or by having a 20% increase over the first year and a steady weight afterwards, or by having a constant increase each month over two years which adds up to 20%. We derived predictions for the latter, i.e., a steady (constant) monthly proportional increase in weight. To end up with a 10% and 20% increase after two years, we thus predicted blood pressure trends at month *x* assuming a proportional weight change of *((1.1)^x/24^-1)* and *((1.2)^x/24^-1),* respectively, at month *x* (Derivation: if the weight of a patient at baseline is *w_b_*, then if we assume a constant monthly increase *r* which adds up to a total of 20% increase after two years, the weight at 2 years can be expressed as *w_24_ = r^24^ w_b_ = 1.2 w_b_ 🡪 r = 1.2^1/24^*. With this, at month *x*, the weight of a patient with a constant monthly increase *r* will thus be *w_x_ = 1.2^x/24^w_b_*. Compared to their baseline weight, the proportional weight change at month *x* will thus be *(1.2^x/24^w_b_ – w_b_)/w_b_ = 1.2^x/24^ -1)*). For binomial mixed models (model B, C, E) we present sample-averaged instead of subject-specific predictions of probabilities/proportions. Subject-specific predictions are derived by prediction for a specific individual with random effect = 0 (can be interpreted as predictions for the “average” subject), while sample-averaged predictions are derived by integration over the distribution of random effects (correspond to “group effects”/differences between groups of subjects).
